# A Rare Presentation of Basal Cell Carcinoma Arising within Trichoepithelioma: A Diagnostic Challenge

**DOI:** 10.7759/cureus.5401

**Published:** 2019-08-16

**Authors:** Tanya Greywal, Ashley G Rubin, Brian Jiang

**Affiliations:** 1 Dermatology, University of California San Diego, La Jolla, USA; 2 Mohs Surgery, Bernardo Dermatology Medical Group, Poway, USA

**Keywords:** basal cell carcinoma, immunohistochemistry, diagnosis, treatment, trichoepithelioma

## Abstract

Differentiating between trichoepithelioma and basal cell carcinoma (BCC) is sometimes diagnostically challenging. We present a case of a 61-year-old male with a BCC arising within a trichoepithelioma, which is rarely reported in the literature. Clinical and histological diagnosis of trichoepithelioma is sometimes complicated by its many similarities to BCC. Therefore, immunohistochemical analysis and adequate tissue sampling are essential in suspicious lesions. In addition, as represented by our patient’s presentation, it is important for clinicians to remember that the presence of a concurrent malignant neoplasm may be masked by the benign nature of a trichoepithelioma and that a superficial shave biopsy may not be sufficient for accurately diagnosing such suspicious lesions.

## Introduction

Trichoepithelioma is a benign hair follicle tumor with many of its clinical and histologic features similar to those of basal cell carcinoma (BCC). Thus, differentiating between these two entities is sometimes diagnostically challenging. We present a case of a 61-year-old male with a BCC arising within a trichoepithelioma, which is rarely reported in the literature (Poster and Abstract: Greywal T, Rubin A, Jiang SB. Basal Cell Carcinoma Arising within Trichoepithelioma: A Rare Presentation and Diagnostic Challenge. American Academy of Dermatology 76th Annual Meeting; Feb 16-20, 2018).

## Case presentation

**Figure 1:**
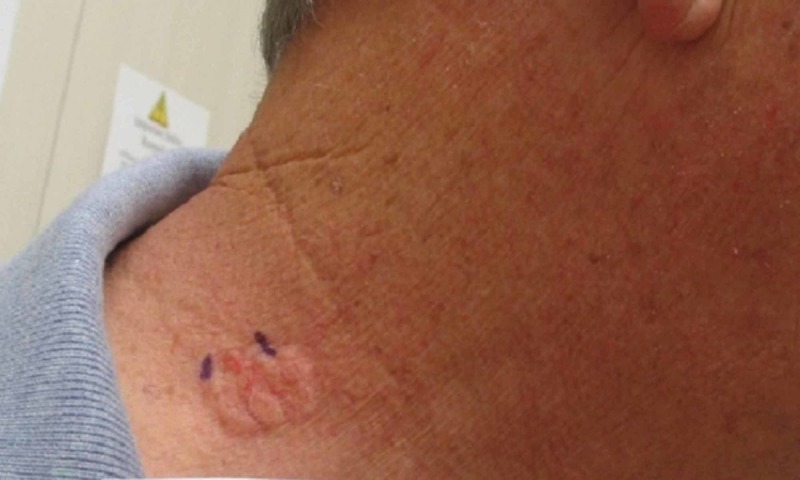
Clinical presentation A 1.5 cm by 2.3-cm yellow, multilobular plaque with overlying telangiectasias on the patient’s neck (purple ink markings define, in part, the margins of the lesion)

A 61-year-old man with no previous history of any type of skin cancer presented with a “spot” on his neck that had been present for 30 years. The lesion slowly increased in size and was associated with intermittent swelling. Cutaneous examination of the neck revealed a 1.5 cm by 2.3-cm multilobular plaque with overlying telangiectasias (Figure 1).

**Figure 2:**
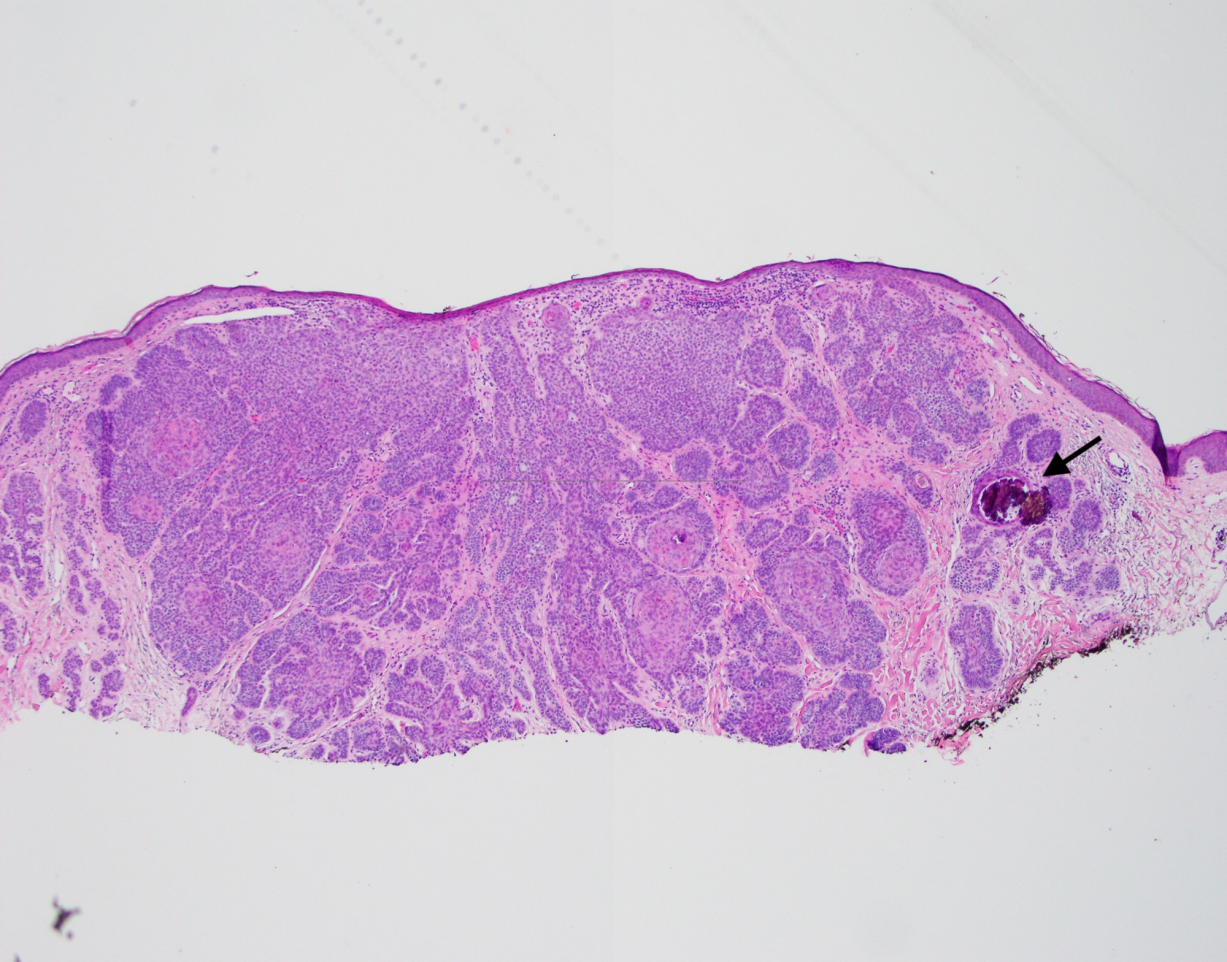
Lower power view of skin biopsy Low power view (4x) showing a multi-lobular basaloid neoplasm with focal dystrophic calcification (arrow).

**Figure 3:**
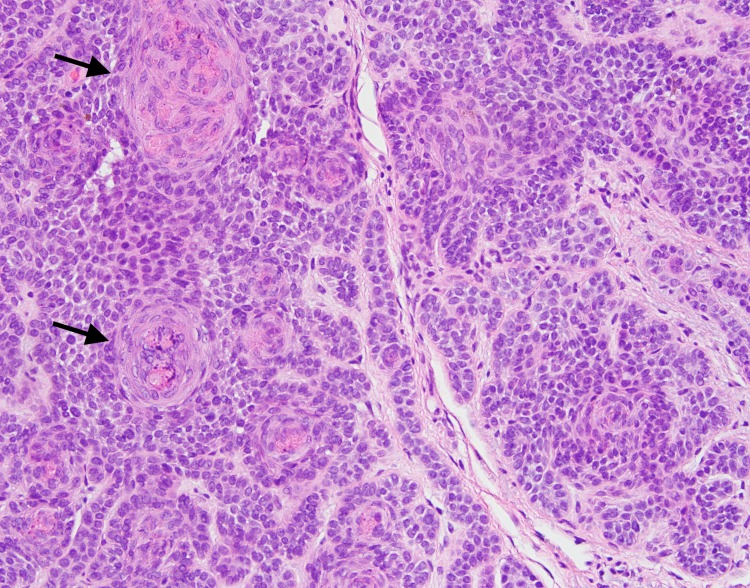
High power view of skin biopsy High power view (20x) showing primitive follicular differentiation (arrows)

**Figure 4:**
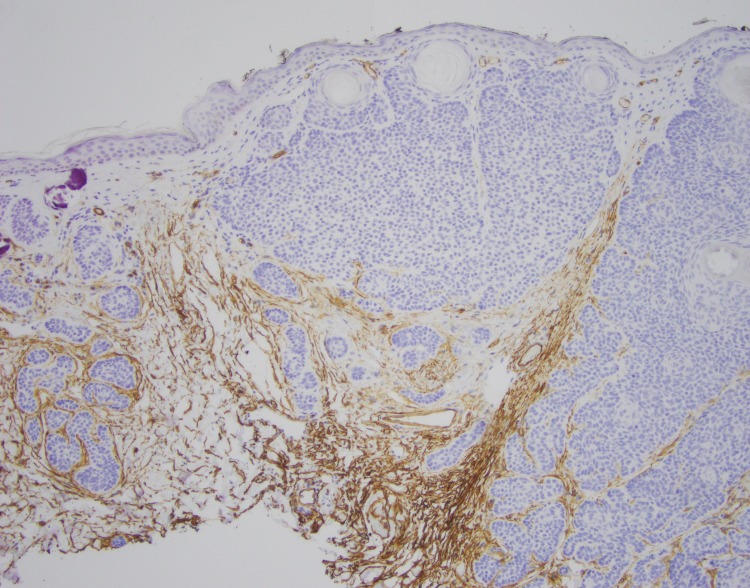
CD34 immunohistochemistry of skin biopsy CD34 immunohistochemistry (brown stain) showing intimate CD34 positive fibroblastic component, which supports the diagnosis of trichoepithelioma

**Figure 5:**
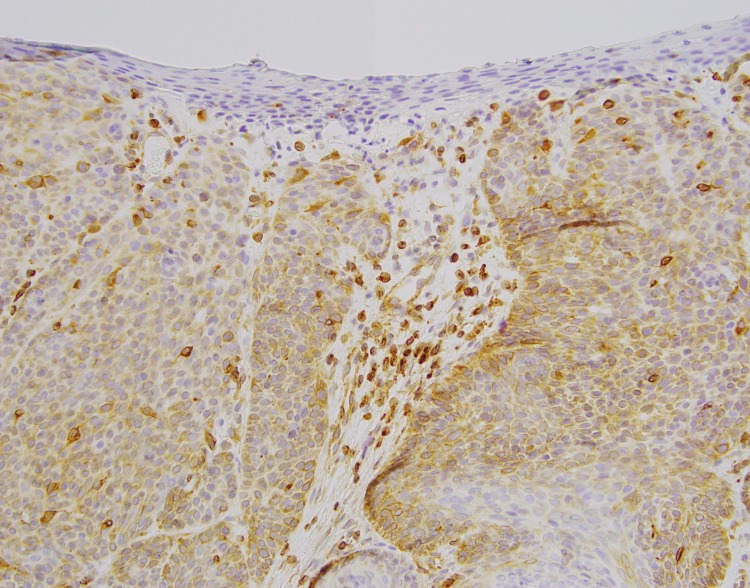
Bcl-2 immunohistochemistry Bcl-2 immunohistochemistry demonstrating decoration of individual tumor cells along the periphery (as seen in trichoepithelioma) and with some areas of more diffuse expression (as seen in BCC)

A skin biopsy of the lesion was performed (Figures 2-5).

Histopathologic examination revealed basaloid islands within a cellular stroma, follicular differentiation with papillary mesenchymal bodies, minimal atypia and apoptotic bodies, and no retraction artifact. Immunohistochemical staining was positive for CD34 in stromal cells and Bcl-2 in the peripheral cells of the basaloid islands; CK20 was negative. These findings were most consistent with trichoepithelioma. However, due to the overlapping histologic features with BCCs, a conservative surgical excision was performed to completely remove the lesion.

**Figure 6:**
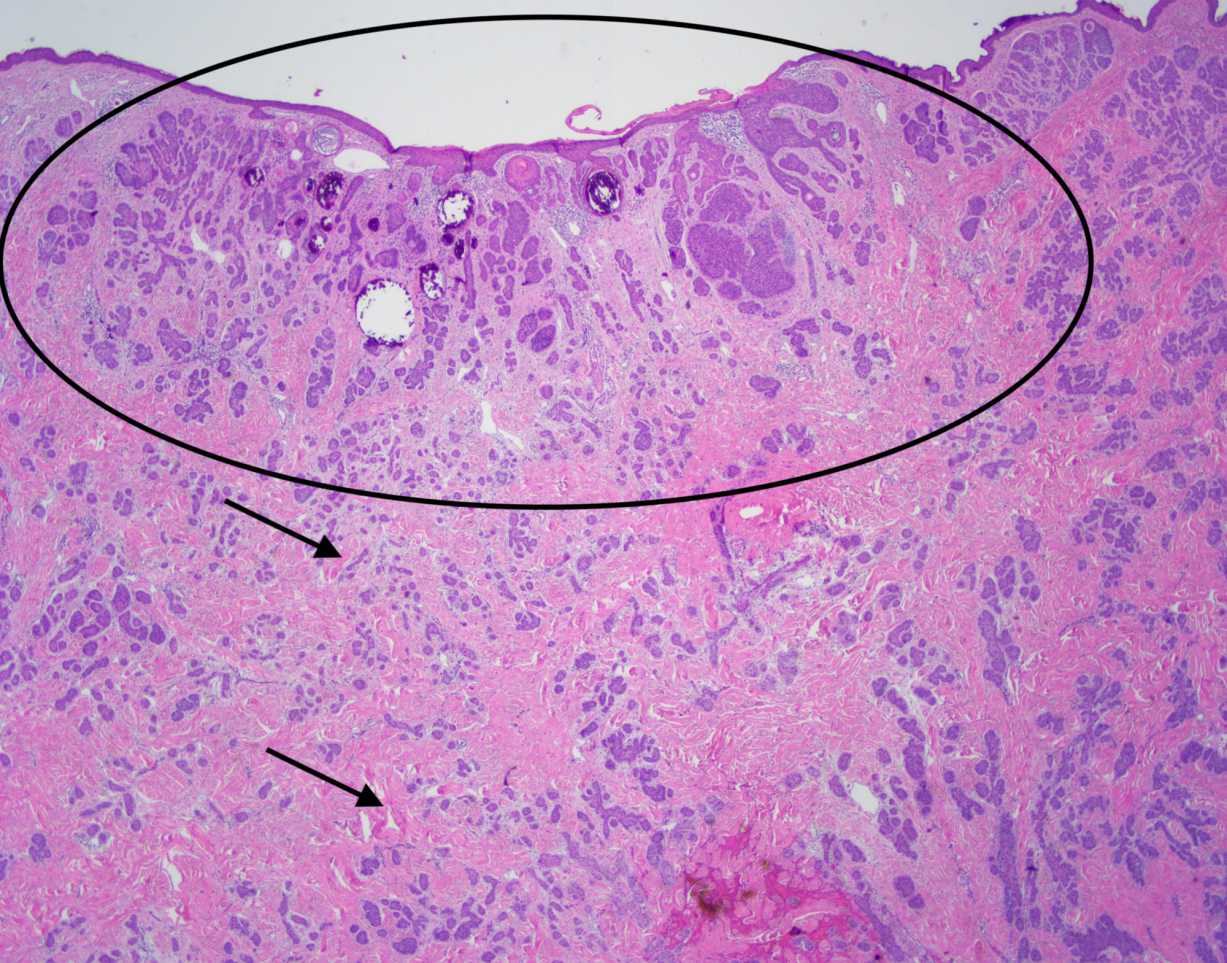
Excisional biopsy Excisional biopsy showing superficial trichoepitheliod neoplasm (circle) with underlying diffusely infiltrative basal cell carcinoma (arrows)

**Figure 7:**
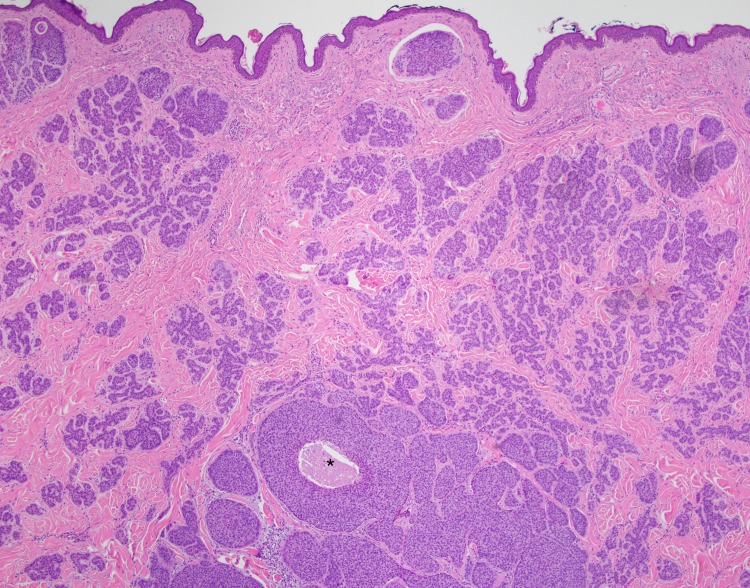
Excisional biopsy, low power view Low power view (4x) of invasive basal cell carcinoma with focal cystic degeneration (asterisk)

**Figure 8:**
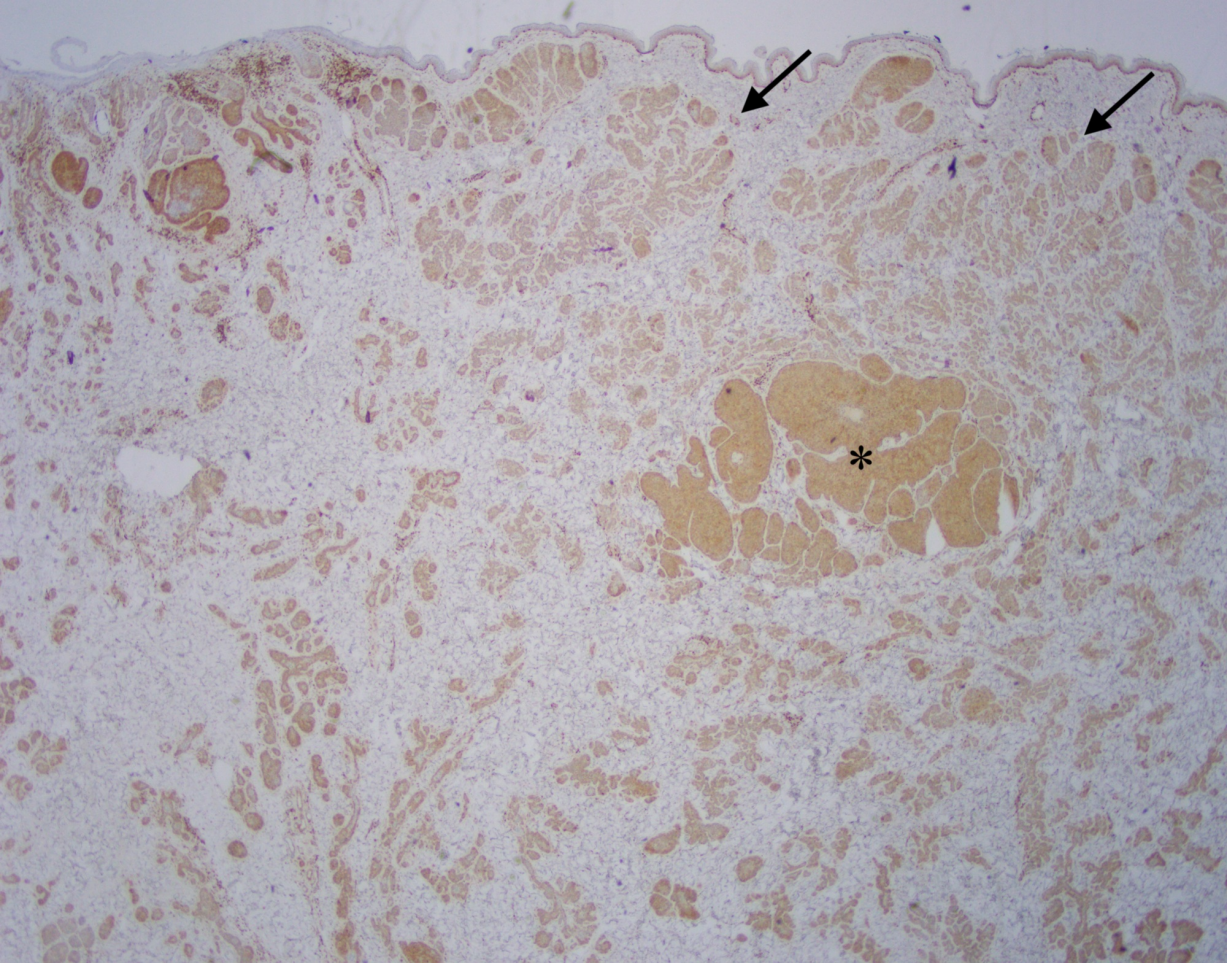
Excisional biopsy, Bcl-2 immunohistochemistry Bcl-2 staining with prominent expression in tumor cells. Note the staining pattern throughout the invasive BCC component (asterisk), and primarily along the periphery of the trichoepithelioma (arrows).

**Figure 9:**
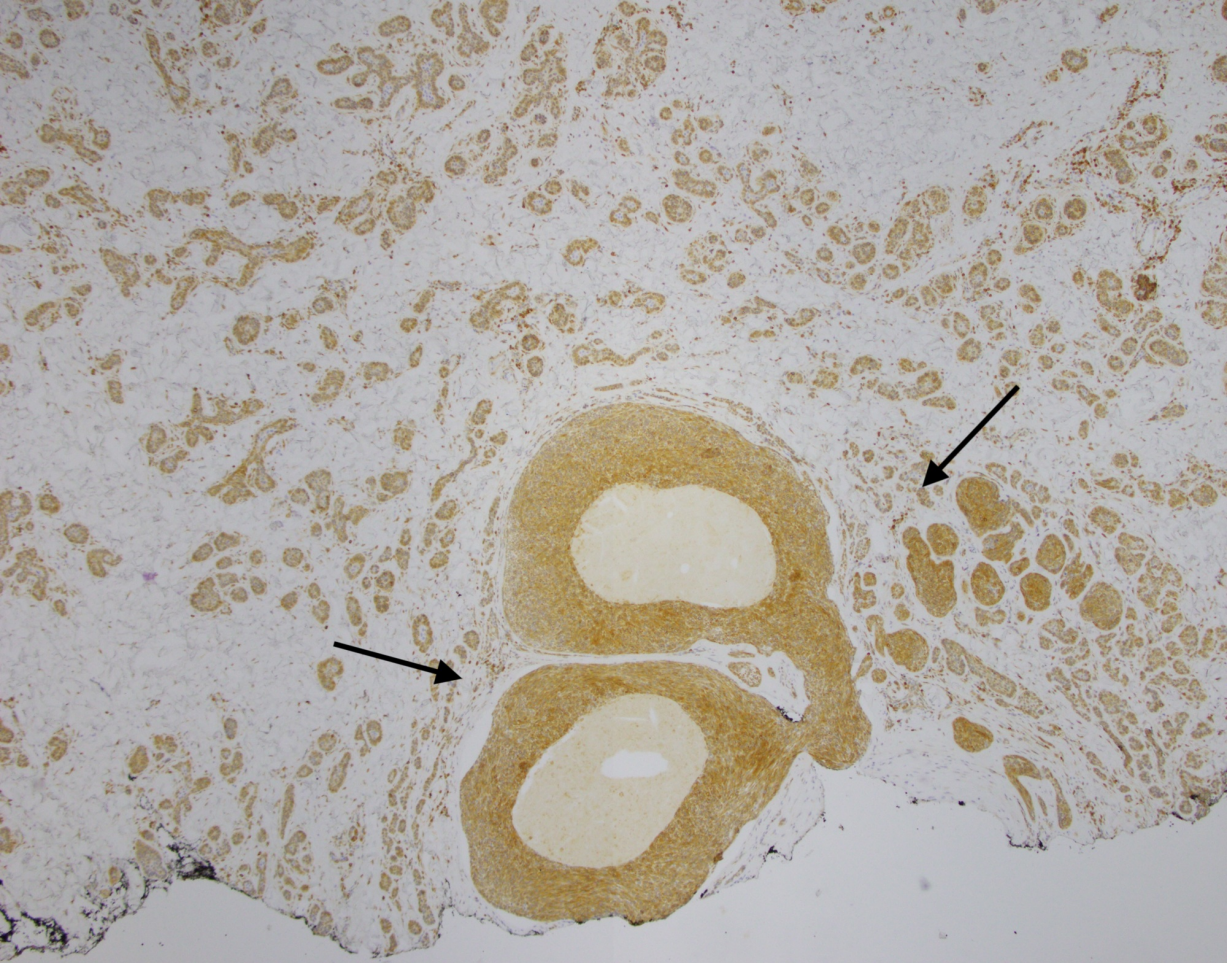
Excisional biopsy, low power view, Bcl-2 immunohistochemistry Low power (4x) Bcl-2 stain with decoration of the majority of component basaloid cells consistent with BCC (arrows) BCC, basal cell carcinoma

**Figure 10:**
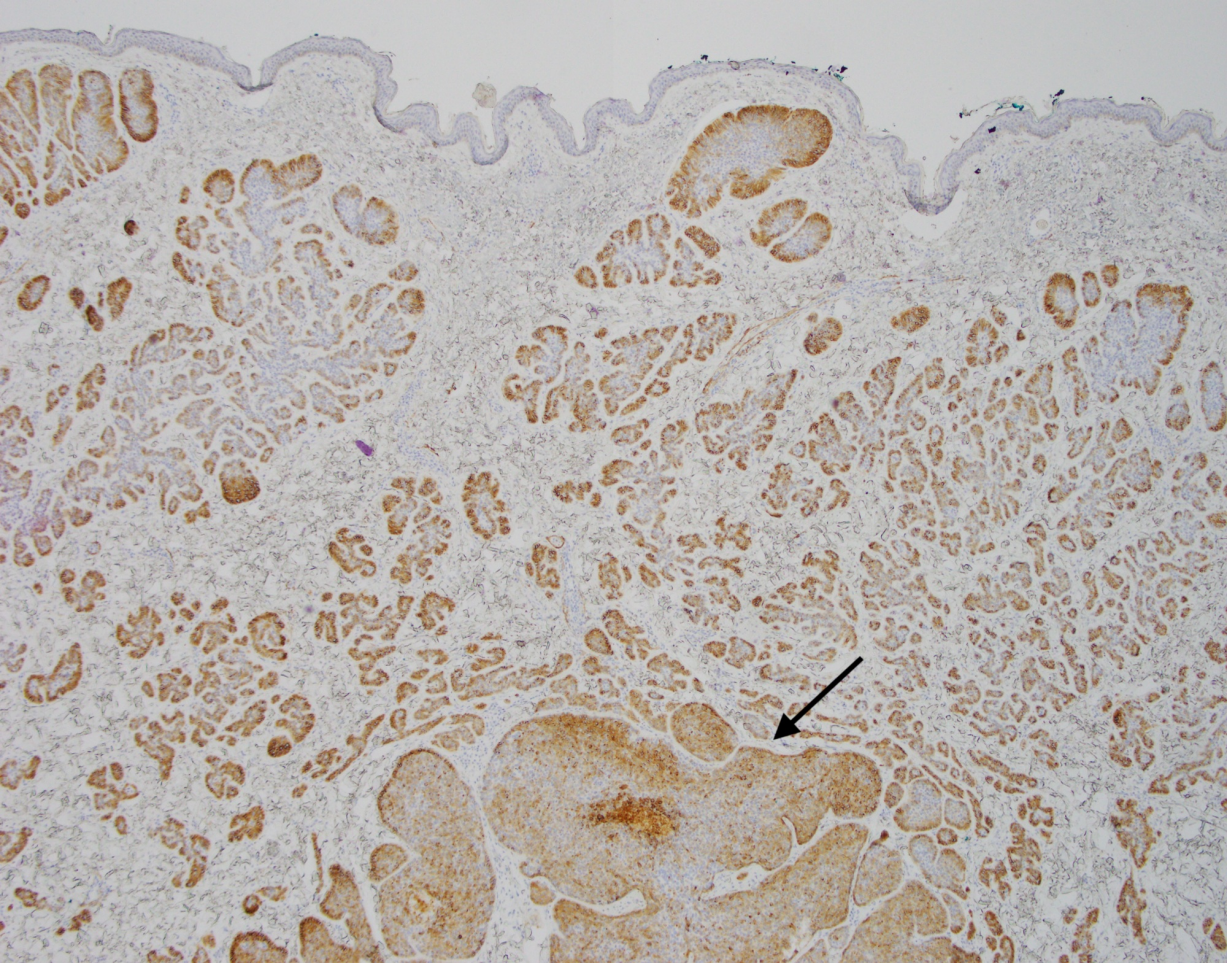
Excisional biopsy, CD10 immunohistochemistry Low power view (4x) showing CD10 decoration of component BCC tumor cells (arrow) BCC, basal cell carcinoma

**Figure 11:**
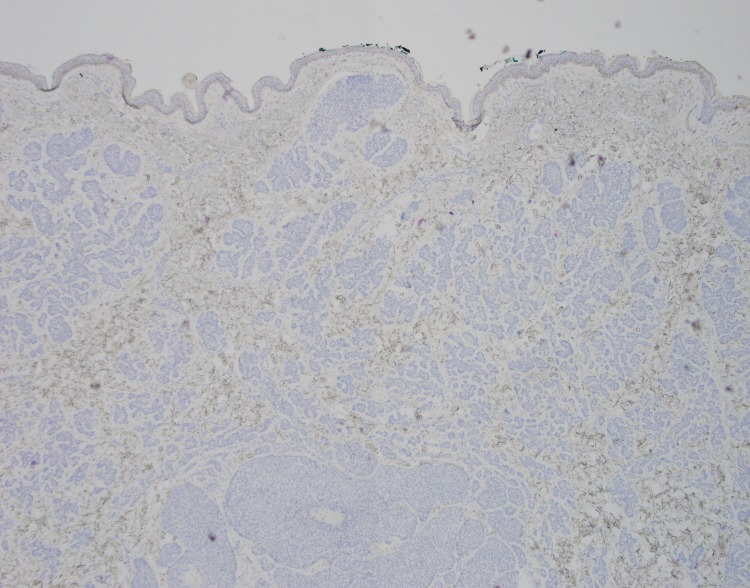
Excisional biopsy, CK20 immunohistochemistry Negative CK20 stain with no significant expression within the more superficial trichoepithelioma or deeper BCC BCC, basal cell carcinoma

Histopathologic examination of the excised tissue revealed multiple basaloid nodules (Figures 6-11).

Some of the larger nodules contained cystic formations, necrosis, peripheral retraction artifact, and increased atypia; the smaller nodules showed follicular differentiation. Immunohistochemical staining was diffusely positive for Bcl-2 and CD10 in these larger nodular and cystic areas, and peripherally positive in areas with follicular differentiation. CK20 remained negative in all components. Overall, these findings were most consistent with a multifocal, infundibulocystic basal cell carcinoma arising within a trichoepithelioma. Since the lesion extended broadly into the deep margins, re-excision was recommended.

**Figure 12:**
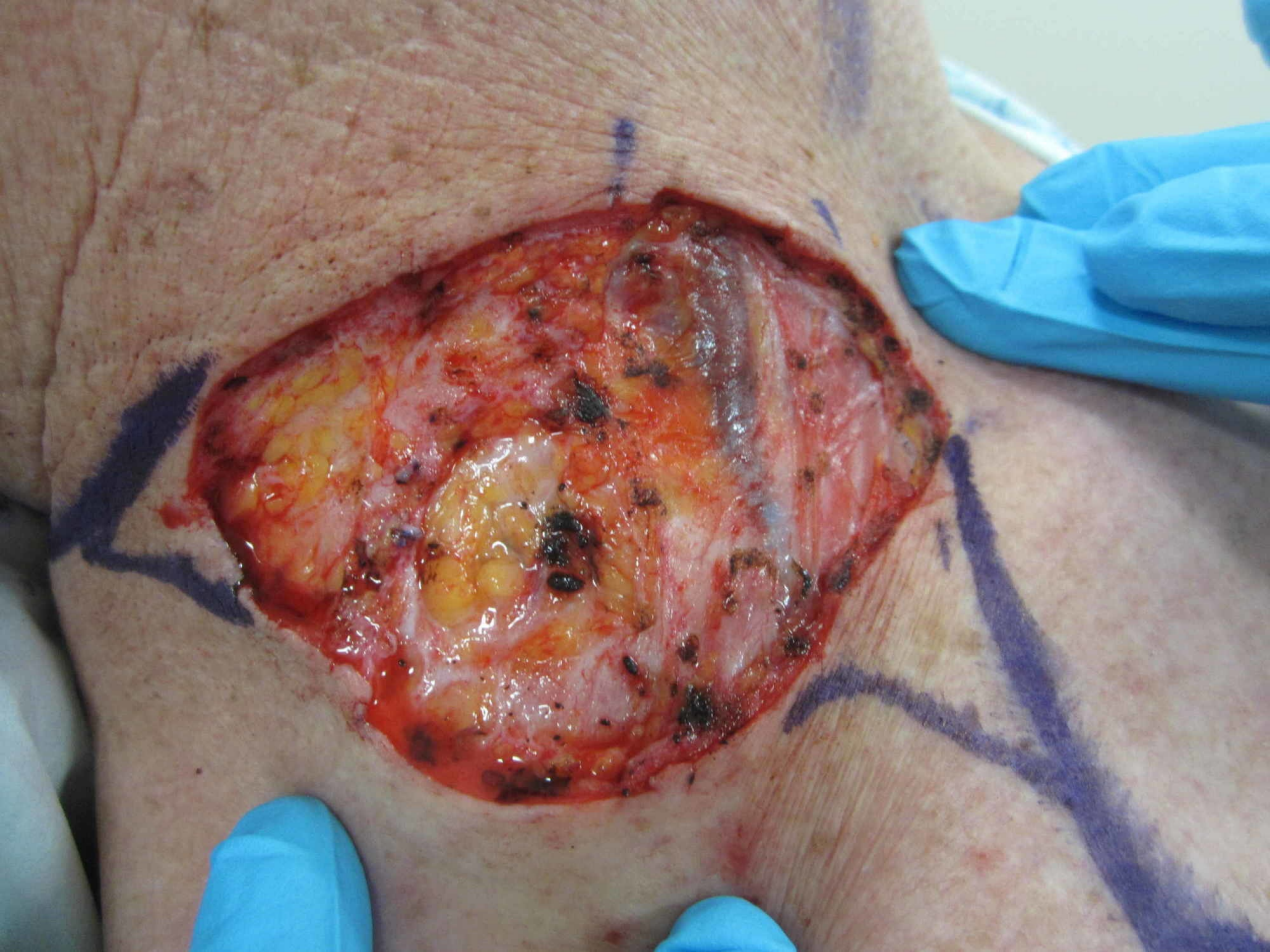
Final Mohs surgery defect The final defect after four stages of Mohs micrographic surgery (7.2 cm by 8.0 cm)

The patient subsequently underwent a third and final procedure with Mohs micrographic surgery (MMS) to achieve optimal clearance of the margins. The final defect was 7.2 cm by 8.0 cm after four stages of MMS (Figure 12).

Following confirmation of negative margins, the resultant wound was repaired with an advancement flap.

## Discussion

It is difficult to differentiate trichoepithelioma from BCC due to their many clinical and histologic similarities (Table 1).

**Table 1:** Clinical, histopathologic, and immunohistochemical features of trichoepithelioma and basal cell carcinoma

	Trichoepithelioma	Basal Cell Carcinoma
Clinical Features	- Typically presents as a skin-colored papule or nodule in children and young adults	- Most common non-melanoma skin cancer - Pearly skin-colored papule with telangiectasias on sun-exposed areas in older individuals
Histopathologic Features	- Islands of basaloid cells that do not interact with the epidermis - Papillary mesenchymal bodies - Horn cysts - Fibroblastic stroma - No high-grade atypia - Few to no mitoses	- Basaloid islands that may connect with the epidermis - Clefting between tumor and stroma - Peripheral palisading of basaloid cells - Central cell necrosis - Myxoid stroma - Mitotic figures
Immunohistochemical Features	Bcl-2: peripheral epithelial expression CD10: stains stromal cells, especially around the tumor cells CD34: stains fibroblastic stroma around basaloid cells	Bcl-2: diffuse expression CD10: stains stromal and tumor cells CD34: no expression

Trichoepitheliomas are rare, benign neoplasms with three histologic variants: desmoplastic, multiple, and solitary [1-4]. These tumors are thought to originate from follicular germinative cells and result from epithelial-mesenchymal origin cell proliferation [2,5]. Trichoepitheliomas can develop in any ethnicity or gender [1]. Males and females are equally affected, although some believe that women have a greater predilection for trichoepithelioma in inherited conditions, since men have lower levels of penetrance and expressivity [1-2]. By contrast, BCCs are the most common non-melanoma skin cancer that can originate from the epidermis or outer root sheath of a hair follicle; it has several histologic subtypes and is most prevalent in males [6-7].

Trichoepitheliomas usually present as a skin-colored papule or nodule on the central face of children and young adults [1-4,8]. They may also develop in other less common areas such as the neck, scalp, and trunk [1,4]. In addition, telangiectasia and central depression may be detected within these lesions [1,4,8]. BCC has a similar clinical appearance and is typically found on the head, neck, and other chronically sun-exposed areas in older individuals [6]. BCCs can present as large lesions, unlike trichoepitheliomas that grow slowly over time and typically range from 2 mm to 8 mm in size [2]. Our patient’s lesion represents an unusual presentation of trichoepithelioma since this neoplasm rarely exceeds 1cm in size [4,8].

The diagnosis of trichoepithelioma is classically based upon histologic examination. However, accurate diagnosis is complicated by the many histologic similarities between trichoepithelioma and BCC. Trichoepitheliomas and BCCs both present with nests of basaloid cells with follicular differentiation. Trichoepitheliomas are characterized by islands of basaloid cells that do not interact with the epidermis, papillary mesenchymal bodies, horn cysts, fibroblastic stroma, no high-grade atypia, few or no mitoses, monomorphic nuclei, abortive hair papillae, and occasional calcium deposits [1-4,8-11]. BCCs display basaloid islands that may connect with the epidermis, clefts between the tumor and stroma (retraction artifact), peripheral palisading of basaloid cells, cell necrosis, epidermal ulceration, myxoid stroma, and mitotic figures [8,10-11].

Immunohistochemistry is important in differentiating trichoepitheliomas from BCCs. Clinicians typically use Bcl-2, CD10, CD34, and CK20 immunohistochemical stains to aid in diagnosis. Bcl-2 is an anti-apoptotic protein that is diffusely expressed in BCCs, and only stains peripheral epithelial cells and papillary mesenchymal bodies in trichoepitheliomas [11-12]. In trichoepitheliomas, CD10 only stains stromal cells, particularly those surrounding tumor cells; conversely, CD10 stains both the stromal and tumor cells in BCCs and is classically positive along the periphery of the tumor [5,13]. Positive CD34 staining of the fibroblastic stroma surrounding nests of basaloid cells is present in trichoepithelioma and absent in BCC [2,11]. CK20 highlights Merkel cells, which are typically present in trichoepitheliomas and rarely found in BCCs [5,12,14-15]. However, several studies question the efficacy of CK20 in differentiating trichoepithelioma and BCC, since the quantity of Merkel cells varies between their many histologic subtypes of trichoepitheliomas [12,14-15]. In addition, since this marker highlights only scattered, single cells, it may not be reliable, especially when evaluating a small amount of tissue from a superficial shave biopsy [15].

Several studies have investigated the patterns of other immunohistochemical stains such as cytokeratin 19 (CK19), debrin, nestin, laminin-5, and PHLDA1. CK19 is expressed in germinative basaloid cells and can positively stain both BCCs and trichoepitheliomas [10]. However, one study evaluated patterns of CK19 staining and found diffuse, focal, and negative staining in 60%, 28%, and 12% of BCC cases, respectively [10]. They also showed diffuse, focal, and negative staining in 12%, 29%, and 59% of trichoepithelioma cases, respectively [10]. Therefore, CK19 will most likely diffusely stain BCCs, and remain negative in trichoepitheliomas. Debrin is an F-actin binding protein that appears to be highly and homogenously positive in BCCs and weakly present in a non-homogenous distribution within trichoepitheliomas [5]. Nestin is expressed in mesenchymal cells in hair follicles, and variable expression of nestin in trichoepitheliomas and BCCs is reported in the literature [11,16]. Stromal cells are usually positive in trichoepitheliomas and negative in BCCs [16]. However, certain histologic subtypes of BCC (nodular, superficial, infiltrative) show variable, weak nestin expression in their stromal cells [15]. Laminin-5 (laminin-5γ2 chain) was studied and shown to be positive in 96.2% of BCCs and in only 12% of trichoepitheliomas [11,17].

Finally, PHLDA1 (also known as TDAG51) is the most recent immunohistochemical stain that has been investigated for its role in differentiating trichoepithelioma and BCC. PHLDA1 is a proline- and glutamine-rich protein involved in apoptosis regulation and is expressed in melanocytes, the basal layer of the follicular bulge, and the lowermost portions of the inner root sheath and catagen follicles [12,14]. PHLDA1 is diffusely positive in trichoepithelioma and negative in BCC (although it may be positive near superficial ulcerations) [14-15]. Thus, some suggest PHLDA1 should replace CK20 since it can more easily and consistently differentiate trichoepithelioma and BCC, even in small biopsy specimens [14-15].

Since trichoepithelioma and BCC possess many similar features, it is important to realize that a superficial shave biopsy may provide inadequate tissue sampling for definitive diagnosis. Therefore, an excisional biopsy may be recommended in order to obtain more lesional tissue and to allow for a more accurate diagnosis.

Some cases have been reported in the literature describing the concomitant presence of a trichoepithelioma and a BCC, as seen in our patient [9,11,18-20]. Most of these cases presented patients with both trichoepithelioma and BCC in the setting of genetic syndromes such as Brooke-Spiegler syndrome, familial cylindromatosis, or multiple familial trichoepithelioma [9,18-19]. These conditions are all due to germline mutations of the cylindromatosis (CYLD) gene on chromosome 16q12-q13, which lead to uncontrolled activity of NF-κB, and ultimately, cell proliferation [1,8-9].

Other case reports discuss the possibility of malignant transformation of trichoepithelioma to BCC, which usually arises in patients with multiple trichoepitheliomas [11,20]. Although trichoepitheliomas are generally thought to be benign neoplasms, they can rarely undergo malignant transformation, which is important for clinicians to consider when making diagnostic and therapeutic decisions [11,19-20]. In addition, the coexistence of trichoepithelioma and BCC may be the result of two neoplasms (a collision tumor) independently developing in the same location [11,20]. Our patient illustrates a unique presentation of a BCC arising within a trichoepithelioma.

The treatment of trichoepithelioma includes laser, cryotherapy, dermal abrasion, electrodessication and curettage, radiation, and surgery [1-3]. Surgical removal can safely remove the lesion and is a particularly important modality when concomitant neoplasms or malignant transformation is suspected [2].

## Conclusions

Trichoepithelioma is a tumor that seldom grows beyond 1 cm in size and rarely undergoes malignant transformation. However, clinical and histological diagnosis of trichoepithelioma is sometimes complicated by its many similarities to BCC. As such, immunohistochemical analysis and adequate tissue sampling are essential in suspicious lesions. Our patient presented with a BCC arising within a large, atypical trichoepithelioma. Therefore, it is important for clinicians to remember that trichoepitheliomas may co-exist with malignant neoplasms and that a superficial shave biopsy may not be sufficient for accurately diagnosing suspicious lesions.
